# Molecular and functional characterization of GAD67-expressing, newborn granule cells in mouse dentate gyrus

**DOI:** 10.3389/fncir.2013.00060

**Published:** 2013-04-02

**Authors:** Carolina Cabezas, Theano Irinopoulou, Bruno Cauli, Jean Christophe Poncer

**Affiliations:** ^1^INSERM, UMR-S 839Paris, France; ^2^Université Pierre et Marie CurieParis, France; ^3^Institut du Fer à MoulinParis, France; ^4^CNRS UMR-S 7102, Laboratoire de Neurobiologie des Processus AdaptatifsParis, France

**Keywords:** granule cells, dentate gyrus, adult neurogenesis, GAD, GABA

## Abstract

Dentate gyrus granule cells (GCs) have been suggested to synthesize both GABA and glutamate immediately after birth and under pathological conditions in the adult. Expression of the GABA synthesizing enzyme GAD67 by GCs during the first few weeks of postnatal development may then allow for transient GABA synthesis and synaptic release from these cells. Here, using the GAD67-EGFP transgenic strain G42, we explored the phenotype of GAD67-expressing GCs in the mouse dentate gyrus. We report a transient, GAD67-driven EGFP expression in differentiating GCs throughout ontogenesis. EGFP expression correlates with the expression of GAD and molecular markers of GABA release and uptake in 2–4 weeks post-mitotic GCs. These rather immature cells are able to fire action potentials (APs) and are synaptically integrated in the hippocampal network. Yet they show physiological properties that differentiate them from mature GCs. Finally, GAD67-expressing GCs express a specific complement of GABAA receptor subunits as well as distinctive features of synaptic and tonic GABA signaling. Our results reveal that GAD67 expression in dentate gyrus GCs is a transient marker of late differentiation that persists throughout life and the G42 strain may be used to visualize newborn GCs at a specific, well-defined differentiation stage.

## Introduction

Dentate gyrus granule cells (GCs) convey synaptic signals from entorhinal cortex to the hilus and Ammon's horn through a complex axonal arborization, the mossy fibers (MFs). Although GCs are primarily glutamatergic neurons, mossy fiber terminals (MFTs) have been shown to contain GABA (Sandler and Smith, 1991) and glutamate decarboxylase (GAD) (Sloviter et al., 1996). This was observed both in juvenile animals (Gutierrez et al., 2003) and in the epileptic dentate gyrus in adults (Sloviter et al., 1996). Thus, GCs appear to contain two, fast-acting neurotransmitters with opposing post-synaptic effects and may switch neurotransmitter modality during postnatal developmental and in pathological conditions (Gutierrez, 2005). Consistent with this hypothesis, putative monosynaptic IPSCs were recorded in CA3 principal cells upon *minimal* MF stimulation (Walker et al., 2001; Gutierrez et al., 2003; Safiulina et al., 2006), suggestive of synaptic GABA release from MFTs and activation of post-synaptic GABAA receptors. However, this conclusion was recently challenged by experimental evidence showing that (1) the vesicular transporter of GABA (VIAAT) is not detected in MFTs (Sperk et al., 2003; Uchigashima et al., 2007), (2) monosynaptic IPSCs evoked by extracellular stimulation in the granule cell layer do not show typical properties of synaptic transmission from MFTs (Uchigashima et al., 2007), (3) stimulation of individual GCs does not induce monosynaptic IPSCs in CA3 pyramidal cells (Mori et al., 2004; Cabezas et al., 2012).

One difficulty in studying dentate gyrus GCs arise from their delayed and heterogeneous maturation. More than 80% GCs differentiate during the first few weeks after birth (Altman and Bayer, 1990) and neurogenesis persists in the subgranular zone of the dentate gyrus throughout life (Kempermann et al., 2004). Thus, extracellular stimulation, particularly during the first weeks of postnatal life, is likely to recruit MFs from a highly heterogeneous population of GCs with regard to their differentiation stage. The reported timing of GAD67 expression in dentate gyrus GCs [up to 3 weeks after birth, (Gutierrez et al., 2003)] strikingly coincides with that of intense postnatal neurogenesis, suggesting GAD67 may be expressed specifically in differentiating GCs. Further supporting this hypothesis, enhanced neurogenesis and GAD67 expression in dentate gyrus GCs have been observed in the epileptic hippocampus (Sloviter et al., 1996; Gutierrez et al., 2003; Jessberger et al., 2007; Parent, 2007).

In order to explore the expression of GAD67 in GCs, we used a GAD67-EGFP BAC transgenic mouse strain (Chattopadhyaya et al., 2004). In the hippocampus, EGFP expression was strikingly restricted to a population of dentate gyrus GCs. By combining electrophysiological recordings, immunohistochemistry and single cell RT-PCR, we show that GAD67 expression in MFTs is restricted to a subpopulation of immature GCs both in juvenile and adult hippocampus. These cells are 2–4 weeks postmitotic cells, express GABAergic markers and are synaptically integrated in the hippocampal network. Yet they differ from mature GCs in many respects including their intrinsic and synaptic properties. Our results, together with previous observations (Cabezas et al., 2012) suggest GAD67 expression is a transient phenotype of differentiating GCs and GABA signaling in these cells differ from that in fully mature GCs.

## Materials and methods

### Animals

Design of G42 transgenic mice has been described previously (Chattopadhyaya et al., 2004). Mice were continuously backcrossed on a C57BL/6 background, and only males were used for experiments. Although hydrocephalus has been reported for this mouse strain (http://jaxmice.jax.org/strain/007677.html), we found that its incidence was very much reduced by breeding hemizygous mice to C57BL/6. In the very few cases in which hydrocephalus was observed, animals were not used for experiments. All the procedures carried out in this study were performed in accordance with the European Communities Council Directive (86/809/EEC) on the care and use of animals for experimental procedures and was approved by local ethical committees.

### Immunohistochemistry

Mice were rapidly anesthetized by intraperitoneal injection of ketamine/xylazine (80/20 mg/kg, Sigma–Aldrich) and perfused transcardially with 4% (w/v) paraformaldehyde in 0.1 M sodium phosphate buffer, pH 7.5. Brains were post-fixed overnight in the same solution at 4°C and cryoprotected in 30% sucrose for an additional 48 h. Transverse, 40 μm-thick sections were cut with a cryotome (Microm KS34, Thermo Scientific) and stored at −20°C in a solution containing 30% (v/v) ethylene glycol, 30% (v/v) glycerol, and 0.1 M sodium phosphate buffer, until they were processed for immunofluorescence as follows. *Day 1*, free-floating sections were rinsed 3 times for 15 min in PBS. They were then incubated for 1 h in PBS supplemented with 0.3% Triton X-100 and 5% normal goat serum. Finally, they were incubated overnight at 4°C with primary antibodies. The following antibodies were used: GFP (polyclonal chicken, 1:1000, Chemicon); Prox1 (polyclonal rabbit, 1:10000, Chemicon); Doublecortin (Dcx polyclonal rabbit, 1:2000; Cell Signaling); Calretinin (monoclonal mouse, 1:5000, Swant), Calbindin D-28K (monoclonal mouse IgG1, 1:1000, Swant); NeuN (monoclonal mouse, 1:250, Chemicon); VIAAT (polyclonal rabbit, 1:1000, kindly provided by Dr. Bruno Gasnier). *Day 2, s*ections were rinsed three times for 15 min in PBS and incubated for 1 hour with goat Cy3-coupled (1:600) and/or goat Cy5-coupled (1:400), and/or donkey-FITC (1:500; all from Jackson Laboratory) secondary antibodies. Sections were rinsed for 10 min twice in PBS and mounted with Mowiol/Dabco (25 mg/ml) and stored at 4°C before imaging.

For bromodeoxyurudine labeling, two groups of G42 mice were given two bromodeoxyuridine (BrdU) i.p. injections (300 mg/kg in 0.9% saline, Sigma–Aldrich) 3 h apart. At days 1, 8, 15, 21, 28, and 35 after injections, the brains were perfused, fixed and cut as described above. Sections were washed twice in PBS, incubated in 2N HCl (30 min at 37°C), and rinsed in 0.1 M borate buffer, pH 8.4 (10 min). Sections were incubated in PBS with 0.4% Triton X-100 and 5% normal goat serum for 30 min, followed by overnight incubation with primary anti-BrdU antibody (monoclonal rat, 1:200; AbD Serotec). After rinsing, sections were incubated overnight with primary anti-GFP antibody (polyclonal chicken, 1:1000, Chemicon). Sections were then incubated for 1 h at room temperature with secondary Ab; goat anti-rat-Cy3 (1:600, Jackson labs) and donkey anti-chicken-FITC (1:500, Jackson Labs), then washed and mounted in Mowiol/Dabco (25 mg/ml).

### Confocal imaging and analysis

Double- and triple-labeled images were obtained using sequential laser-scanning confocal microscopy (SP2 or SP5; Leica Microsystems) using 40X/1.25 N.A and 63X/1.32 N.A objectives. Typically, stacks of 10–40 sections 0.5 or 1 μm apart were acquired (depending on the experiment). Confocal images were analyzed with Imaris 6.0 (Bitplane) and MetaMorph (Molecular Devices) softwares. For co-localization data, cells were counted as immunopositive when their fluorescence level equaled at least twice the background level and co-localization was estimated from each confocal plane of a stack.

For estimating the proportion of EGFP+ GCs, the entire DG was imaged in 3D in at least 3 different slices from each animal. We estimated the volume of individual GCs somata from the mean value of ten cells. The number of cells in the entire DG was estimated by dividing the volume of the granule cell layer by the mean volume of a cell. The EGFP+ GCs were counted manually.

### Electrophysiology

Horizontal hippocampal slices were prepared from postnatal day 10–60 G42 mice. Mice were anesthetized by intraperitoneal injection of ketamine and xylazine (80 and 20 mg/kg, respectively) and decapitated. The brain was quickly removed and immersed in low-sodium, ice-cold artificial CSF (low Na^+^-ACSF) equilibrated with 95% O_2_–5% CO_2_. The composition of the low Na^+^-ACSF was (in mM): 248 sucrose, 26 NaHCO_3_, 10 glucose, 5 MgCl_2_, 4 KCl, 1 CaCl_2_, and 5 ppm phenol red. 350 μm thick slices were prepared using a vibroslicer (Microm HM650V, Thermo Scientific) and incubated at room temperature in ACSF saturated with 95% O_2_–5% CO_2_. ACSF was composed of (in mM): 124 NaCl, 26.2 NaHCO_3_, 11 D-glucose, 2.5 KCl, 1 NaH_2_PO_4_, 3 CaCl_2_, and 2 MgCl_2_. For recording, slices were transferred to a submerged chamber maintained at 31°C, mounted on an upright microscope (Olympus BX51WI) equipped with infrared illumination, and superfused with ACSF at a rate of 2–2.5 ml/min. For electrophysiological characterization of DG GCs, whole-cell recordings were made under visual guidance, using patch electrodes (3–5 MΩ resistance) made from borosilicate glass capillaries (Hilgenberg) and filled with (in mM): 120 KMeSO_3_, 8 KCl, 10 HEPES, 5 EGTA, 3 MgCl_2_ (pH 7.4, 280–290 mOsm). Spontaneous activity was recorded at −70 mV, from both EGFP-positive and negative GCs. Spontaneous post-synaptic currents (spPSCs) were analyzed with Detectivent software (Ankri et al., 1994). Signals were acquired and filtered at 10 kHz using and Multiclamp amplifier and digitized at 20 kHz using the Clampex software (Molecular Devices). Access resistance was monitored online. All parameters included in the study (see Table 1) were analyzed offline with Clampfit 10 and Detectivent. The decay time constant of spontaneous IPSCs (Figure 7) was calculated from an average of 150–200 events aligned on their rising phase, using Detectivent software. The initial 30 ms of the current decay from the peak was fit to a monoexponential equation of the form *f(t)* = Ae^−t/τ^ +C, in order to minimize contamination by other, spontaneous IPSCs. Mean PSC frequencies were derived from the mean of instantaneous, inter-event intervals.

**Table 1:** **Compared electrophysiological properties of GAD67/EGFP expressing and non-expressing GCs**.

	**RMP (mV)**	**Cm (pF)**	**Threshold (pA)**	**AP width (ms)**
Group 1/EGFP+ (*n* = 32)	−63.9 ± 2.4	38.4 ± 3.9	62.4 ± 14.3	3.8 ± 0.4
Group 1/EGFP− (*n* = 33)	−66.2 ± 2.6 ns	85.4 ± 10.8^*^	72.8 ± 11.7 ns	2.7 ± 0.2 ns
Group 2 (*n* = 22)	−80.2 ± 1.4^*^^,^ ^##^	132.0 ± 22.9^*^^,^ ^#^	95.0 ± 5.7^*^^,^ ^#^	1.4 ± 0.1^*^^,^ ^##^

*^#^Comparison with EGFP−*.

Mann–Whitney rank sum test significance

* p < 0.001, for comparisons with Group 1/EGFP+ GCs and

# p <0.05,

## *p <0.001 for comparison with Group 1/EGFP− cells*.

For tonic current measurements, intracellular solution was composed of (in mM): 135 CsCl, 10 HEPES, 10 EGTA, 4 MgATP (2H_2_O), 0.4 NaGTP (2H_2_O), 1.8 MgCl_2_ (pH 7.4, 280–290 mOsm). Cells were recorded at −70 mV, in the presence of DL-APV (100 μM), NBQX (10 μM). To study the differential expression of δ-containing GABA_A_ receptors, after 5–10 min of control recording, the high affinity agonist, 4,5,6,7-tetrahydroisoxazolo[5,4-c]pyridin-3-ol (also known as gaboxadol, GBX, 2 μM), was added to the ACSF. At the end of the experiment, bicuculline (40 μM) was added to the solution. The mean baseline current was determined by fitting a Gaussian distribution to the histogram obtained from six epochs of 3000 points each, taken every 25–30 s. The mean of the fitted Gaussian was considered to be the mean holding current. In a given neuron, we derived the amplitude of GBX-induced tonic current by subtracting the mean holding current in control vs. in the presence of GBX.

### Cytoplasm harvest and scRT-PCR

In some experiments, at the end of the recording, the cytoplasm of the cell was harvested in the recording pipette. After cytoplasm collection, the patch pipette was gently withdrawn to allow the closure of the cell membrane (Cauli et al., 1997) and its content was expelled into a test tube, and RT was performed in a final volume of 10 μ l as described previously (Lambolez et al., 1992). The scRT-PCR protocol was designed to detect simultaneously the expression of 32 different genes (Table A1). Two amplification steps were performed essentially as described previously (Cauli et al., 1997). Briefly, the cDNAs present in 10 μ l of the RT reaction were first amplified simultaneously by using the primer pairs listed in Table A1 (except for EGFP all primer pairs were intron overspanning). Because EGFP amplified sequence is intronless, a negative control for genomic DNA contamination (amplifying the SST gene intron) was always included to ascertain the mRNA origin of the EGFP amplified product (Hill et al., 2007). *Taq* polymerase (2.5 U; Qiagen) and 20 pmol of each primer were added to the buffer supplied by the manufacturer (final volume, 100 μl), and 21 cycles (94°C for 30 s, 60°C for 30 s, and 72°C for 35 s) of PCR were run. Second rounds of amplification were performed using 1 μ l of the first PCR product as template. In this second round, each cDNA was amplified individually with a second set of a primer pair internal to the pair used in the first PCR (nested primers) (Table A1). Thirty-five PCR cycles were performed as described previously. Then 10 μl of each individual PCR product was run on a 2% agarose gel using ϕX174 digested by *Hae*III as molecular weight maker and stained with SYBR Safe DNA gel stain. All the transcripts were detected from 500 pg of neocortical RNA using this protocol (data not shown). The sizes of the PCR-generated fragments were as predicted by the mRNA sequences (Table A1).

### Unsupervised clustering

To classify cells, unsupervised clustering was performed using 13 electrophysiological features (input and access resistance, membrane capacitance and time constant, resting membrane potential, firing threshold (in mV), amplitude and half width of the first action potential, amplitude of the AHP after the first action potential, number and mean interval of action potentials (APs) during a 150 pA/500 ms current step, amplitude of the last action potential and firing adaptation index), 32 molecular parameters (see Table A1), EGFP presence or absence (visually detected during recording) and the age the animals from which cells were recorded. Neurons positive for GAD65 and/or GAD67 were denoted as GAD positive and their mRNAs were considered as a single molecular variable as described previously (Karagiannis et al., 2009). Parameters were standardized by centering and reducing all values. Cluster analysis was run on Statistica 6 software (Statsoft).

In Ward's method (Ward, 1963), individual cells are first linked to their nearest neighbor and combined two-by-two into objects of a superior hierarchic order. This linkage procedure is repeated on these objects until the top hierarchic level is reached. The final number of clusters was suggested by the Thorndike procedure. Briefly, the average within-cluster distance is plotted at each stage of the amalgamation schedule, resulting in a decrease in the average within-cluster distance as the number of clusters increases. The final number of classes (or cell types) is determined at the stage where the maximal decrease is reached in this plot (Thorndike, 1953).

Comparison of the occurrence of a given molecular marker between populations of cortical neurons was done according to the following equation: |ε| = |*p*a − *p*b|/√[(*pq*/*n*a) + (*pq*/*n*b)]; where *p*a and *p*b represent the percentage of occurrence and *n*a and *n*b the number of individuals in populations a and b, respectively. The variable *p* denotes the percentage of occurrence in the overall population with *q* = 1 − *p*. The parameter ε was tested against a normal distribution to determine statistical significance of the difference of occurrence (Fisher and Yates, 1963).

Averaged data are expressed as mean ± sem. Statistical significance was assessed using nonparametric unpaired test (Mann–Whitney rank sum test), using SigmaStat 3.0 (SPSS).

## Results

### GAD67-driven EGFP expression in differentiating dentate gyrus granule cells

**Figure 1:**
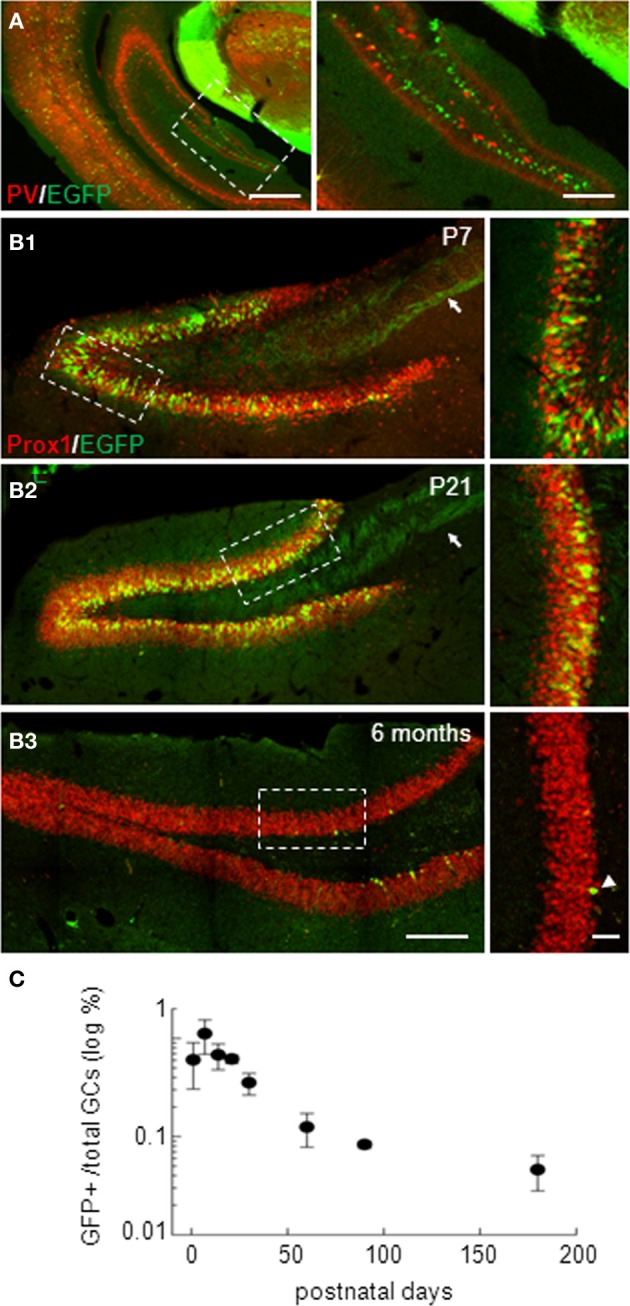
**Dentate gyrus EGFP-positive neurons in G42 mice are mostly granule cells. (A)** Widefield fluorescence images of the isocortex and hippocampus of G42 mice at P30. EGFP (green) colocalizes with parvalbumin (PV, red) in neocortical interneurons. However, in the hippocampus, EGFP expression is restricted mostly to a subpopulation of dentate gyrus neurons and does not colocalize with PV staining. Scale: right, 500 μm; left, 50 μm. **(B)** Confocal maximum projection images showing EGFP expression in the dentate gyrus of P7, P21, and 6 months-old mice. Dentate gyrus granule cells (GCs) are stained with Prox-1, a specific marker of GCs, showing colocalization with EGFP at all post-natal ages. Note the mossy fiber track revealed by EGFP immunoreactivity (arrow). EGFP expression persists in the adult, although in fewer cells (arrowheads), mostly confined near the subgranular zone. Boxed areas in right panels are shown enlarged in left panels. Scale: left, 200 μm; right, 50 μm. **(C)** Estimation of the proportion of GCs expressing EGFP showing a progressive decrease with age, reaching a steady-state in the adult. *n* ≥ 3 slices/animal and 3 animals/age.

In isocortex, G42 mice have been reported to express EGFP specifically in a subpopulation of parvalbumin-expressing GABAergic interneurons (Chattopadhyaya et al., 2004). However, in the hippocampus, EGFP expression was largely confined to the dentate gyrus (Cabezas et al., 2012) where it did not colocalize with parvalbumin (Figure 1A). Instead, EGFP-expressing (EGFP+) cells resembled GCs, as confirmed by immuno-labeling with the GC specific marker Prox-1. We observed a 99.8% colocalization of EGFP and Prox-1 staining at all postnatal ages tested (from P1 to P180, Figures 1B1–B3; *n* ≥ 3 animals/age). We quantified the density of EGFP+ GCs which peaked around the second to third postnatal week. At this stage, EGFP+ mossy fiber track was clearly visible and projected to the CA3 region (arrows in Figures 1B1,B2). The estimated proportion of EGFP+ GCs then decreased gradually with age yet persisting in adults (Figure 1C). Interestingly, EGFP+ cells in older animals were more confined to the inner part of granule cell layer close to the subgranular zone (Figure 1B). This progressive, age-dependent decrease in EGFP+ GCs proportion and confinement to the subgranular zone suggests these neurons might be recently differentiated neurons.

**Figure 2:**
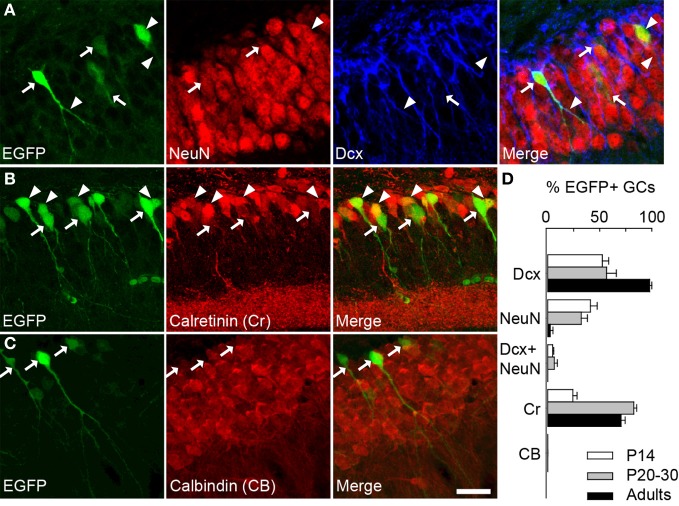
**GAD67-driven EGFP expression is restricted to immature granule cells in G42 dentate gyrus. (A)** Maximum intensity projections of confocal images of hippocampus from P20–30 G42 mice, showing EGFP-positive GCs colocalize primarily with DCx as well as NeuN. Arrowheads indicate co-localization with EGFP whereas arrows indicate lack of co-localization. **(B)** Most EGFP-positive GCs express calretinin (Cr), another marker of immature GCs. Arrowheads indicate co-localization of EGFP with calretinin whereas arrows indicate lack of calretinin immunoreactivity in EGFP+ cells. **(C)** In contrast, no expression of calbindin (CB) was detected in EGFP-positive GCs (arrows). Scale: 20 μm. **(D),** Summary data of developmental marker quantification in juvenile (P14, P20–30) and adults animals (2–6 months) respectively. *n* ≥ 6 slices/animal; *n* ≥ 3 animals/age.

We therefore examined colocalization of EGFP with other markers of GC differentiation at three postnatal ages (P14, 20–30 and adults). Specifically, we examined expression of doublecortin (DCx, expressed in <30 days post-mitotic neurons), NeuN (specific of mature neurons), calretinin (Cr, expressed in immature GCs from second to 3rd week) and calbindin (CB, specific of >30 days post-mitotic GCs), which have been used classically to characterize differentiating GCs (Kempermann et al., 2004; Lledo et al., 2006). In P20–30 mice, more than half EGFP+ cells were also immunopositive for Dcx (56, 6%; Figures 2A,D). Some EGFP+ neurons also expressed NeuN (32.3%) or both NeuN and Dcx (6.3%). These results confirm that EGFP+ neurons in G42 hippocampus are immature GCs, yet likely near the end of their differentiation process. This was also supported by colocalization with Cr (82.7%; Figures 2B,D) but not with CB (Figures 2C,D). Similar results were obtained in P14 and in adult mice (from 2 to 6 months old). In P14 animals where most GCs still undergo differentiation and maturation (Altman and Bayer, 1990), most EGFP+ GCs were DCx positive (52.5%, Figure 2D) but a significant fraction (41.1%) was positive for NeuN (Figure 2D). In contrast, in adult G42 mice, most EGFP+ GCs expressed DCx and Cr (97.9% and 70.5%, respectively, Figure 2D) whereas little or no colocalization with NeuN or CB was observed. Thus, GAD67-driven EGFP expression in dentate gyrus GCs seems to be restricted to a well-defined differentiation stage both in juvenile and adult animals, although greater variability is found in juveniles likely owing to late postnatal development of the dentate gyrus.

**Figure 3:**
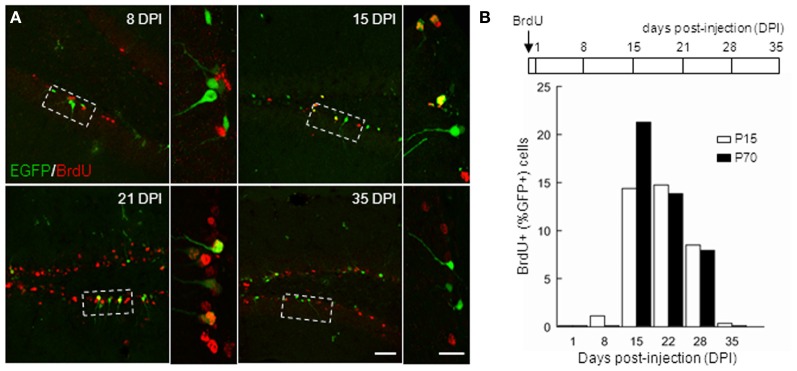
**Timing of GAD67-driven EGFP expression in differentiating granule cells.** P15 and P70 G42 mice received intraperitoneal injection of BrdU and were sacrificed at increasing times after injection. After fixation, brain slices were processed for BrdU and EGFP immunostaining. **(A)** Colocalization of BrdU and EGFP immunoreactivity in maximum intensity projections of confocal stacks reveals EGFP+ neurons born at the time of injection. Boxed areas in left panels are shown enlarged in right panels. Scale 50 μm and 20 μm (insets). **(B)** Top, scheme of BrdU treatment and time course of experiment; Bottom, quantification of data revealing expression of EGFP in approximately 14–28 days postmitotic GCs, both in young and adult mice. *n* ≥ 2 animals/age/day post-injection.

In order to further characterize the time window of GAD67-driven EGFP expression in newborn GCs, we compared EGFP expression in BrdU-injected mice both in juveniles (P14) and adults (P70). We administered BrdU through two i.p. injections of 150 mg/kg at 3 h interval in both groups, and sacrificed the animals at different times (Figure 3B) for EGFP–BrdU colocalization. In P15 animals, EGFP expression was detected from 8 days post injection (dpi), was maximal at 21 dpi and stopped after 35 dpi (Figure 3; *n* ≥ 3 animals/time point). In adult mice, EGFP expression was slightly delayed and was detectable from 15 dpi and stopped also after 35 dpi (Figure 3B). These results, together with the expression profile of other differentiation markers, suggest that GAD67-driven EGFP expression in dentate gyrus GCs is restricted to differentiating GCs, occurs throughout ontogenesis and corresponds to a rather well-defined and constant maturation stage in both juveniles and adults.

### Characterization and classification of GABAergic newborn granule cells

Our results suggest recently born GCs may synthesize GABA during a restricted time window of their differentiation and GAD67 expression may then represent a new marker of late GC differentiation. In a previous study, we demonstrated that EGFP expression in GCs of G42 mice indeed reflects GAD67 expression and that GAD67 protein accumulates in MFTs from EGFP+ GCs (Cabezas et al., 2012). In order to further characterize the physiological and molecular identity of these cells, we compared the passive and active electrical properties as well as gene expression profiles of EGFP+ vs. EGFP− GCs using combined whole cell recordings and multiplex scRT-PCR (Cauli et al., 1997), both in juvenile (P10–20) and young adult (P30–60) G42 mice.

**Figure 4:**
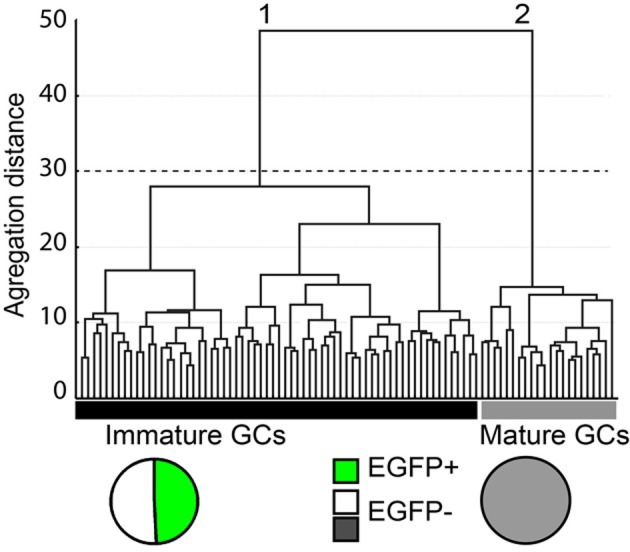
**Cluster analysis of dentate gyrus GCs in G42 mice based on their physiological properties and transcriptome.** *X*-axis, individual cells; *Y*-axis, average within-cluster linkage distance. Two main groups can be distinguished: group 1, including immature GCs, is composed of EGFP+ cells from both juvenile and adult mice (49.2%) and EGFP− cells from juvenile animals (50.8%). Group 2, includes EGFP− GCs from adults. Dotted line indicates the limits between clusters suggested by the Thorndike procedure.

The cytoplasm of a total of 145 GCs was collected after whole cell patch-clamp recording and multiplex RT-PCR was then conducted for 32 genes as described in the *Material and Methods*. Samples showing no Prox-1 expression were excluded from our analysis, so that 87 samples were actually used for unsupervised clustering. In order to identify and define GCs subtypes with consistent yet non-identical properties, we used a polythetic classification scheme (Tyner, 1975) as described (Cauli et al., 2000; Gallopin et al., 2000; Karagiannis et al., 2009). In brief, we used Ward's algorithm (Ward, 1963) to cluster cells with the largest overall similarity and then group these high-order clusters into new ones of decreasing order. A clustering tree (dendrogram) was then derived linking individual cells to higher order groups up to a common root (Figure 4).

All 32 molecular markers as well as 13 electrophysiological parameters, age of the animal and EGFP expression were used for cluster analysis. The hierarchic clustering tree generated by Ward's method showed little difference between all sampled cells, as indicated by the small aggregation distance (Figure 4). However, GCs could be segregated into two main clusters (Figure 4). Group 1 was composed of 65 cells, including all EGFP+ GCs (*n* = 32) from juvenile (16) and young adult groups (16) as well as EGFP− GCs from juvenile group (*n* = 33). This result is consistent with GCs from juvenile animals being still rather immature (Altman and Bayer, 1990). Group 2 was composed of 22 cells, exclusively EGFP− cells from young adult mice (*n* = 20). These results further confirm that GAD67-driven EGFP expression in G42 GCs is specific of recently differentiated neurons that show distinct molecular and physiological properties from fully mature GCs. We next examined in further details the characteristics of these subgroups.

### Electrophysiological properties of GAD67 expressing GCs

Physiological properties of newborn hippocampal GCs have been extensively studied at different stages of differentiation (Ambrogini et al., 2004b; Esposito et al., 2005; Overstreet-Wadiche and Westbrook, 2006; Overstreet-Wadiche et al., 2006). Newborn GCs differ in many respects from mature ones but most strikingly in their inability to fire overshooting APs (Overstreet-Wadiche and Westbrook, 2006). This property is of course critical to predict the impact of these neurons on the hippocampal circuitry and the signals they may convey to their post-synaptic targets. Based on the groups identified in the cluster analysis, we compared the physiological properties of three groups of dentate gyrus GCs: Group 1/EGFP+ cells, Group 1/EGFP− cells and Group 2 cells.

**Figure 5:**
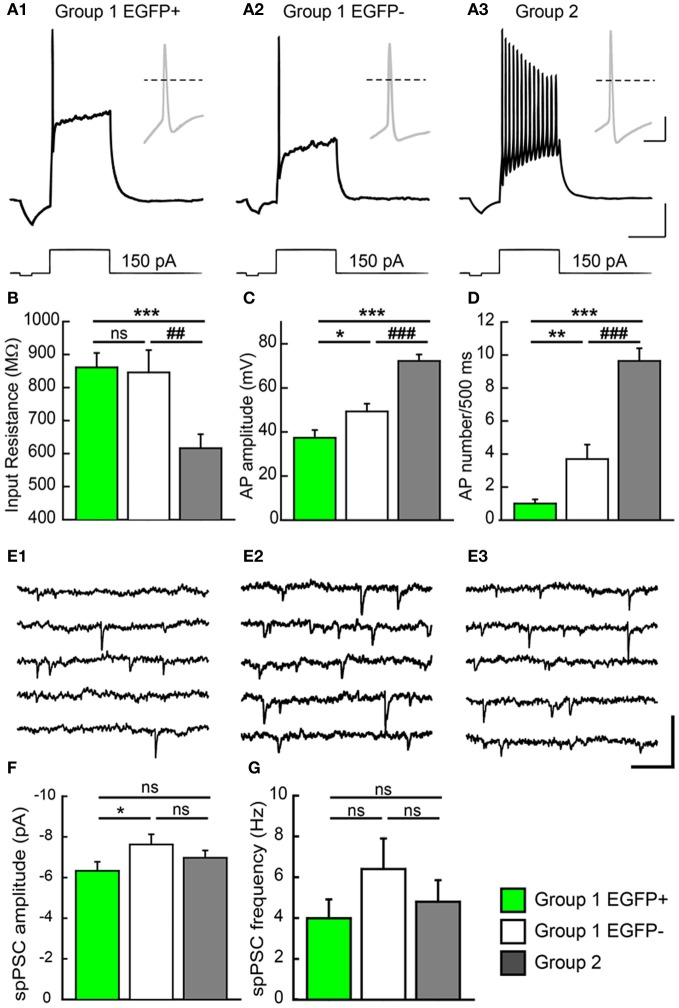
**Electrophysiological properties of GAD67/EGFP expressing and non-expressing GCs. (A)** Compared firing of each GC group identified in the unsupervised cluster analysis: group 1 EGPF+, group 1 EGFP−, and group 2. Response to a +150 pA current step. Insets show the first action potential of the train. Dotted line: 0 mV. Scale: 15 mV, 300 ms (20 mV, 10 ms for insets). **(B)** Summary data of input resistance for all groups (*n* = 32 for group 1 EGFP+, *n* = 33 for group 1 EGFP−, and *n* = 22 for group 2). **(C,D)** Summary data of action potential (AP) amplitudes and frequency (number during a 500 ms pulse) for all groups (*n* = 22 for group 1 EGFP+, *n* = 29 for group 1 EGFP−, and *n* = 22 for group 2). **(E)** Recordings of spontaneous PSCs (spPSCs) at −70 mV in voltage clamp mode, in each subtype of GCs. Scale; 15 pA, 100 ms. **(F,G)** Summary data of spPSC amplitude and frequency, respectively (*n* = 25 for group 1 EGFP+, *n* = 24 for group 1 EGFP−, and *n* = 20 for group 2). Mann–Whitney rank sum test significance ^*^p < 0.05, ^**^p < 0.01, ^***^p < 0.001, for comparisons with group 1 EGFP+ GCs and ^##^*p* < 0.01, ^###^*p* < 0.001 for comparison with group 1 EGFP− cells.

Group 1/EGFP+ GCs showed passive electrical properties similar to those previously reported for immature neurons (Ambrogini et al., 2004a; Overstreet-Wadiche et al., 2006). These cells showed higher input resistance (Rin: 861 ± 44 MΩ, Figure 5B), lower membrane capacitance (Cm: 38.4 ± 3.9 pF; Table 1) and more depolarized resting membrane potential (RMP: −63.9 ± 2.4 mV; Table 1; *n* = 32 in all cases) than Group 2 cells (Rin: 616 ± 42 MΩ, Cm: 132.0 ± 22 pF, and RMP: −80.2 ± 1.4 mV; *p* < 0.001 for all parameters; *n* = 22, Figure 5B and Table 1). However, in contrast to newborn GCs (Overstreet-Wadiche et al., 2006), a majority of EGFP+ GCs (22 out of 32) were able to fire overshooting APs in response to a 150 pA current pulse (Figure 5A). Yet, as compared to Group 2 GCs, AP amplitude in Group 1/EGFP+ cells was smaller (37.4 ± 3.5 vs. 72.3 ± 2.9 mV, *n* = 22 in each group, *p* < 0.001) while AP width was larger (3.8 ± 0.4 vs. 1.4 ± 0.1 ms, *p* < 0.001) and the number of APs during a 500 ms depolarizing pulse was lower (1.45 ± 0.4 vs. 9.6 ± 0.7, *p* < 0.001; Figures 5A–D). In addition, the current threshold for firing of Group 1/EGFP+ GCs was lower than that of Group 2 cells (+62.4 ± 14.3 vs. 95.0 ± 5.7 pA, respectively; *p* < 0.005, Table 1), suggesting EGFP+ cells may require less excitatory drive than fully mature GCs to convey synaptic signals to their post-synaptic targets (Marin-Burgin et al., 2012).

Interestingly, Group 1/EGFP− GCs showed properties intermediate between EGFP+ and mature, Group 2 GCs. Most passive and active properties in these cells were significantly different from those of Group 2 GCs. In particular, they showed intermediate input resistance (845 ± 67 MΩ; *p* > 0.05 and *p* < 0.05 compared to EGFP+ and Group 2 cells, respectively), resting membrane potential (−66.2 ± 2.6 mV; *p* > 0.05 and *p* < 0.001, compared to EGFP+ and Group 2 cells, respectively), firing threshold (72.8 ± 11.7 pA; *p* = 0.5 and *p* < 0.05, compared to EGFP+ and Group 2 cells, respectively) and AP width (2.7 ± 0.3 ms; *p* > 0.05 and *p* < 0.001, compared to EGFP+ and Group 2 cells, respectively, Table 1). Some properties were also statistically different from those of EGFP+ GCs, suggestive of a differentiation continuum Group 1/EGFP+ → Group 1/EGFP− → Group 2. In particular, Group 1/EGFP− GC capacitance (85.4 ± 10 pF), AP amplitude (49.3 ± 3.5 mV) and AP number during a 500 ms, 150 pA current pulse (4.2 ± 0.9) showed intermediate values compared to Group 1/EGFP+ and Group 2 GCs (Table 1).

We recently showed that GAD67 expressing GCs make functional contacts with post-synaptic targets (Cabezas et al., 2012). We asked whether these cells were fully synaptically integrated in the DG network and also received functional synaptic inputs. We thus recorded spontaneous synaptic activity in all three GC groups described above. Spontaneous post-synaptic currents (spPSCs, both glutamatergic and GABAergic) were detected in all EGPF+ GCs (Figure 5E). However, those were of smaller amplitude than those recorded in Group 1/EGFP− GCs (−6.3 ± 0.4 vs. −7.6 ± 0.5 pA; *n* = 25 and 24 cells, respectively; *p* < 0.05, Figure 5F) with no significant difference in mean frequency (3.9 ± 0.9, 6.4 ± 1.5, *p* > 0.05, Figure 5G). Thus, GAD67 expressing GCs, although not fully mature, are able to fire APs, receive synaptic inputs and form synaptic contacts onto their post-synaptic targets. These cells are therefore likely to participate in hippocampal network activity.

### Molecular characterization of GAD67 expressing GCs

**Figure 6:**
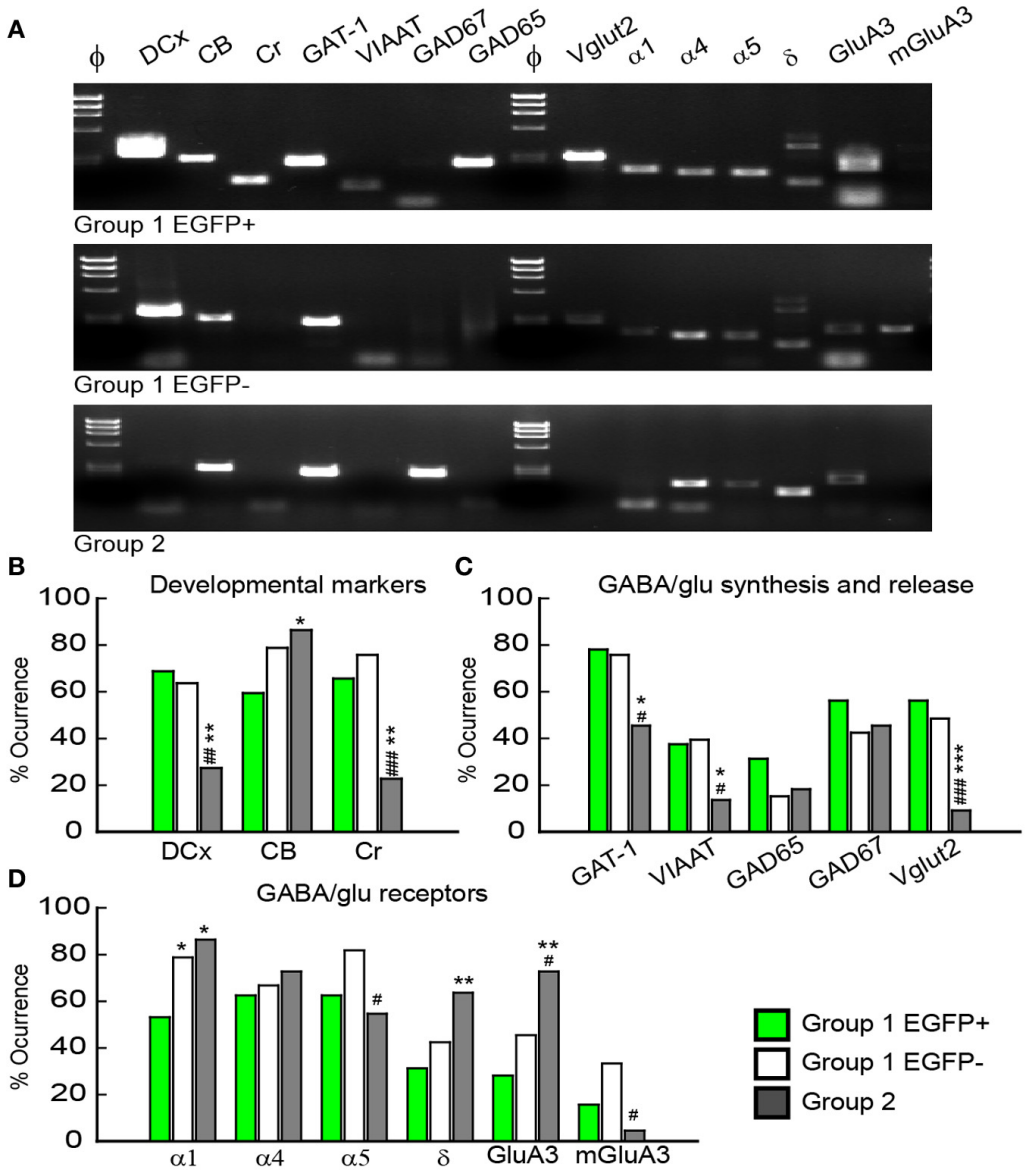
**Molecular characterization of GAD67/EGFP expressing and non-expressing GCs. (A)** Agarose gel analysis of the scPCR products from a representative cell of each GC group (group 1 EGFP+, group 1 EGFP− and group 2). **(B–D)** Quantification of developmental markers (DCX, doublecortin; CB, calbindin; Cr, calretinin), glutamate/GABA synthesis, vesicular release and uptake and subunits of GABAA and glutamate receptors. *n* = 32, 33, and 22 for group 1 EGPF+, group 1 EGFP− and group 2 GCs. Significance ^*^p < 0.05, ^**^p < 0.01, ^***^p < 0.001, for comparisons with group 1 EGFP+ GCs and ^#^*p* < 0.05, ^##^*p* < 0.01, ^###^*p* < 0.001 for comparison with group 1 EGFP− cells.

Multiplex scRT-PCR protocol was designed to detect mRNAs encoding 32 molecular markers (Figure 6A; Table A1) of cellular identity (Prox-1, ZnT3, NPY, TI-VAMP, and GFP), developmental stage (CB, Cr, DCx, and Tub3β), GABA and glutamate metabolism and transport (GAD67, GAD65, VIAAT, Vglut1, Vglut2, GAT-1), chloride homeostasis (KCC2, NKCC1, and CLC-2) and synaptic receptor composition (GABARα1, GABARα4, GABARα5, GABARγ2, GABARδ, NR2A, NR2B, GluA2, GluA3, KA2, GluA6, mGluA2, and mGluA3). We compared the occurrence of these markers among the groups of GCs identified from unsupervised cluster analysis (Table A2).

Consistent with immunohistochemical data, Group 1 (both EGFP+ and −) showed more frequent occurrence of mRNAs for Dcx (66.2%) and Cr (70.8%) than Group 2 GCs (33.8 and 22.7%, respectively, *p* < 0.01), whereas occurrence of CB mRNA was significantly lower (69.2 vs. 86.3%, *p* < 0.05; Figure 6B). Group 1/EGFP+ GCs also expressed mRNAs for GAD65/67, the inhibitory amino-acid vesicular transporter VIAAT as well as the GABA transporter GAT-1 more frequently than mature, Group 2 GCs (Figure 6C). These results are consistent with GABA being synthesized and released from GAD67+ immature but not mature GCs (Cabezas et al., 2012). Yet, GAD-expressing GCs also expressed the glutamate vesicular transporters (both Vglut1 and 2), as expected from neurons primarily releasing glutamate onto all post-synaptic targets (Mori et al., 2004; Cabezas et al., 2012). Interestingly, although the mRNA for Vglut1 was similarly expressed in Group 1 and Group 2 GCs (69.2 vs. 68.2%, respectively, *p* > 0.05), Vglut2 was frequently detected in immature, Group 1 GCs (52.3%) but only very rarely in mature Group 2 GCs (9.1%), suggesting the modalities of glutamate vesicular transport at MFTs may be developmentally regulated.

**Figure 7:**
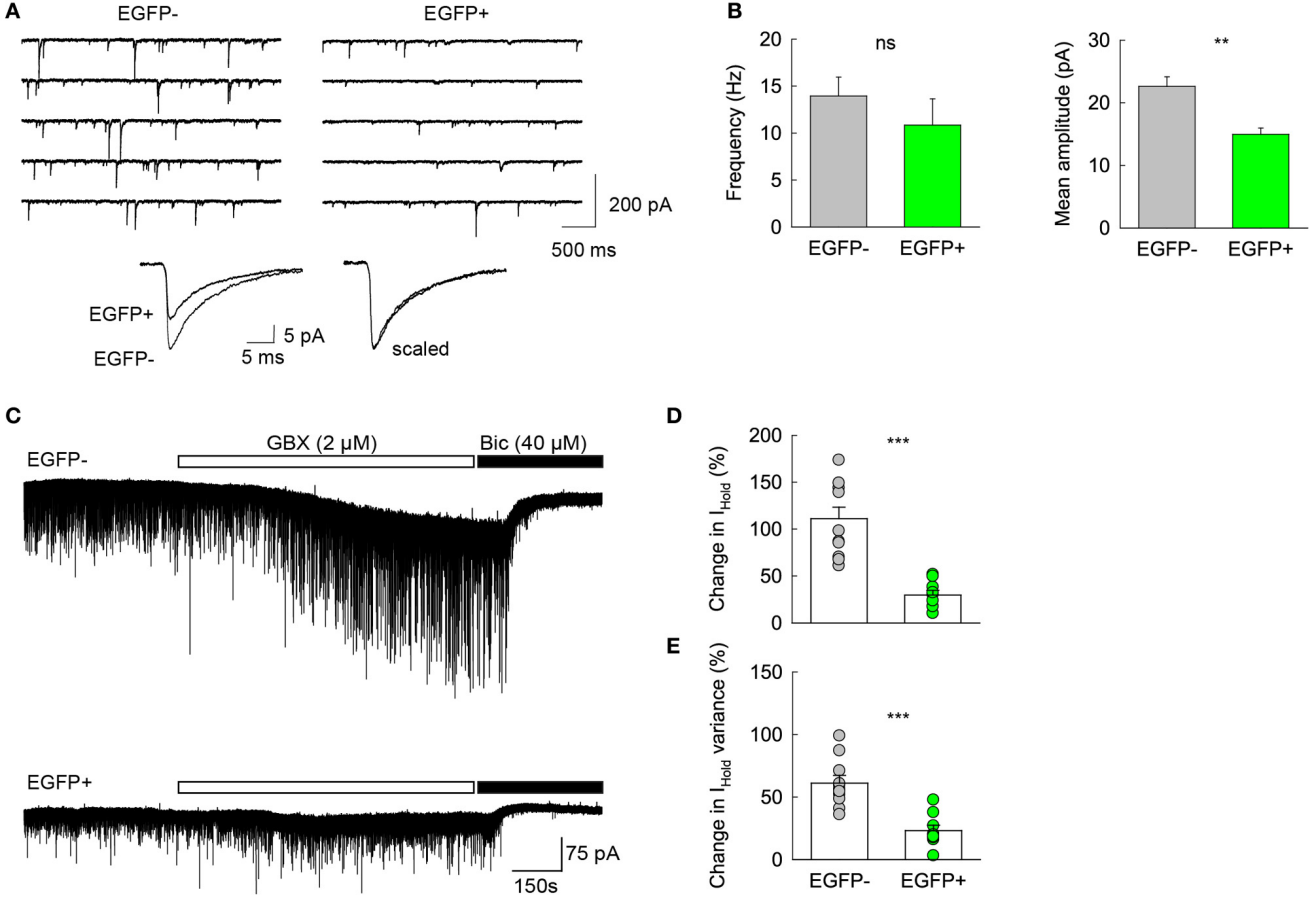
**Differential GABA signaling in GAD67/EGFP expressing vs. non-expressing GCs. (A)** Top spIPSC recordings from Group2/EGFP− and Group1/EGFP+ GCs. Bottom, 150–200 spIPSCs from each recording in top panels were averaged (left) and scaled in peak amplitude (right) to show similar decay kinetics. **(B)** Summary plots of mean IPSC frequency and amplitude from 11 EGFP− and 9 EGFP+ GCs. ^**^p < 0.005. **(C)** Top, recording of a representative EGFP− mature GC from young adult (P35) G42 mouse. 2 μM gaboxadol (GBX) was applied after a 5 min control before bicuculline (40 μM) was added to the recording solution. Bottom, same as above in a representative EGPF+ GC. Note the small effect of GBX in the EGFP+ as compared to that observed in the EGFP− GC, suggesting a lower contribution of δ subunit-containing GABAA receptors. **(D)** Summary data of GBX-induced currents in EGFP+ and EGFP− GCs **(E)** Summary data of GBX effect on the standard deviation of holding current in EGFP− (*n* = 11) and EGFP+ (*n* = 9) GCs. Mann–Whitney rank sum test significance: ^**^p < 0.01, ^***^p < 0.001.

Previous reports have shown that changes in GABAA receptor subunit composition during neonatal development are associated with changes in IPSC kinetics in dentate gyrus GCs (Hollrigel and Soltesz, 1997). Recordings from newborn GCs identified by pro-opiomelanocortin (POMC)-driven EGFP expression or retroviral infection confirmed these cells express distinct complements of GABAA receptor subunits as compared to mature GCs (Overstreet Wadiche et al., 2005; Duveau et al., 2011). In Group 1/EGFP+ GCs, we found a significant yet lower occurrence of the mRNA for the α1 subunit of GABAA receptors than in mature, Group 2 cells (53.1 vs. 86.4%, respectively, *p* < 0.02, Figure 6D). We compared spontaneous IPSC (spIPSC) properties in EGFP+ and EGFP− cells. The mean amplitude of spIPSCs was significantly lower in immature, EGFP+ cells as compared with EGFP− cells (15.0 ± 1.0 vs. 22.6 ± 1.5 pA, *p* < 0.005, *n* = 9 and 11 cells, respectively; Figures 7A,B) with no significant difference in their mean frequency (10.9 ± 2.8 vs. 14.0 ± 2.0 Hz, *p* = 0.2). However, the decay time constant of spIPSCs was not significantly different in EGFP+ as compared with EGFP− cells (8.3 ± 1.0 vs. 6.7 ± 0.9 ms, *p* = 0.2; Figures 7A,B). Thus immature, GAD67-expressing GCs functionally receive less synaptic inhibition than mature GCs but the differential expression of α1 does not translate into detectable changes in IPSC kinetics. In addition to α1, we observed remarkable differences in the expression of GABAA receptor subunits primarily involved in mediating tonic inhibition. In particular, the δ subunit was significantly more frequent in EGFP− than in EGFP+ GCs (63.6 vs. 31.3%, respectively, *p* < 0.02; Figure 6C). In mature GCs, tonic GABAA receptor mediated currents are primarily carried by δ containing receptors (Glykys et al., 2008). Reduced expression of this subunit in immature GCs may then lead to reduced tonic currents in these cells. We tested this hypothesis by comparing tonic GABA currents in EGFP+ and EGFP− expressing neurons in adult (P30–60) G42 mice. In basal conditions, the mean amplitude of tonic GABAA currents was not significantly different in EGFP+ vs. EGFP− GCs (5.6 ± 4.3 vs. 6.5 ± 2.7 pA, *n* = 4 and 7 cells, respectively, *p* = 0.8). We then tested the contribution of δ-containing receptors to tonic GABA currents in both cell groups. To this end, we used the high affinity agonist 4,5,6,7-tetrahydroisoxazolo[5,4-c]pyridin-3-ol (gaboxadol, GBX), at a concentration specific of δ-containing GABAA receptors [2 μM, (Meera et al., 2011)]. GBX increased holding currents in recordings from EGFP− GCs by 111.2 ± 12.1% (*n* = 11; Figures 7C,D). This effect was associated with an increase in the standard deviation of the current by 60.6 ± 5.7% and was entirely blocked by the GABAA receptor antagonist bicuculline (40 μM), consistent with increased GABAA receptor activation (Figure 7E). In contrast, GBX was less efficient in EGFP+ GCs where it increased holding currents by only 29.8 ± 5.0% (*n* = 9; *p* < 0.001, as compared to EGFP− cells, Figures 7C–E). Thus, GABAA receptor subunits contributing to both tonic and synaptic GABA signaling in dentate gyrus GCs are differentially expressed during GC differentiation and receptors mediating tonic inhibition in mature GCs are only weakly expressed in 2–4 weeks post-differentiation GCs.

## Discussion

We have shown that GAD67-driven EGFP expression in G42 mice is restricted to a subpopulation of recently born dentate gyrus GCs. EGFP expression is observed during a well-defined period of GC differentiation time—from 2 to 4 weeks—and this phenotype is maintained with only minor changes throughout postnatal life. Thus, the G42 strain represents a new experimental model allowing direct visualization of a defined state of GC maturation. GAD67/EGFP-expressing GCs show electrophysiological properties typical of not fully mature cells. Yet they are (1) able to fire action overshooting potentials, (2) synaptically integrated in the dentate gyrus network and (3) therefore likely to participate in information flow between cortical structures and hippocampal network. However, they show distinct neurotransmitter modalities and receive less GABAergic inhibition than mature GCs.

### GAD67-driven EGFP expression in G42 mice granule cells

Our results demonstrate that GAD67-driven, EGFP expression in the dentate gyrus of G42 mice is restricted to recently differentiated GCs with only few or no EGFP+ interneurons. This pattern contrasts with isocortex and cerebellum where EGFP expression was reported primarily in parvalbumin-expressing interneurons (Ango et al., 2004; Chattopadhyaya et al., 2004) and with retina (Huckfeldt et al., 2009) and spinal cord (Zhang et al., 2010) where only GABAergic interneurons are labeled. Interestingly, most BAC transgenic mice expressing GFP under the control of the GAD67 promoter show GFP expression in only subsets of GABAergic interneurons, suggesting a complex regulation of GAD67 promoter activity (but see Tamamaki et al., 2003). A recent study using another BAC transgenic strain with GAD67-driven EGFP expression reported a pattern of EGFP expression somewhat similar to that described here, yet with earlier onset and more prolonged expression (Zhao et al., 2010). Whereas the BAC clone in this strain contained 120 kb of upstream and 46 kb downstream sequences, the G42 strain was generated with a BAC clone containing 60 kb of upstream and downstream regions (Chattopadhyaya et al., 2004). Thus, differences in EGFP expression profiles in the two GAD67-EGFP strains may reflect the presence of specific regulatory sequences in the GAD67 BAC clones as well as differences in the locus of BAC recombination (Palmiter and Brinster, 1986).

Despite these differences, we have shown that EGFP expression in G42 GCs does reflect GAD67 expression (Cabezas et al., 2012). GAD67 mRNA was detected both in EGFP+ and EGFP− GCs. However, GAD67 protein immunoreactivity was detected primarily in the MFTs from EGFP+ GCs but not in their somato-dendritic compartments, as described earlier (Sloviter et al., 1996; Gutierrez et al., 2003). In contrast, MFTs from EGFP− GCs were mostly devoid of GAD67 immunoreactivity, even at P15 when a significant fraction of GCs are not fully differentiated (Altman and Bayer, 1990). Thus, although GAD67-driven EGFP expression in G42 hippocampus may not fully mimic endogenous GAD67 expression in GABAergic interneurons, it does reflect GAD67 expression at mRNA and protein levels in a population of dentate gyrus GCs.

### The identity of GAD67-EGFP+ dentate gyrus granule cells in G42 mice

Several transgenic strains have been produced that transiently express fluorescent markers at specific stages of GC differentiation. These strains are particularly useful to characterize the anatomical and functional phenotype of differentiating GCs. Whereas Nestin promoter-driven EGFP expression identifies primarily progenitor cells (Fukuda et al., 2003), doublecortin promoter drives reporter expression over a more prolonged time window that includes both late progenitor stages as well as immature GCs (Couillard-Despres et al., 2006). Mice expressing EGFP under the control of POMC were also shown to transiently express EGFP in newborn dentate gyrus GCs (Overstreet et al., 2004). These cells were immature GCs, likely corresponding to early stage 5 according to the nomenclature of Kempermann and colleagues (Kempermann et al., 2004; Overstreet-Wadiche et al., 2006). GAD67-expressing GCs in the G42 mice appear to be more mature than POMC-EGFP+ cells. First, they express doublecortin, calretinin and NeuN, which are typical markers of immature yet postmitotic GCs. In particular, calretinin and NeuN expression is delayed as compared to doublecortin which is also expressed in progenitor cells (Kempermann et al., 2004). Lack of calbindin expression on the other hand suggests these cells are not fully mature GCs. EGFP+ cells also show electrical and synaptic properties intermediate between early stage 5 cells—as detected by POMC-EGFP expression—and mature GCs. Their input resistance (<1GΩ) and action potential half-width (<4 ms) are intermediate between those of POMC-EGFP+ and mature GCs. GAD67-EGFP+ GCs are synaptically integrated in the hippocampal network and both receive functional synaptic inputs (this study) and form functional synapses onto post-synaptic targets in the hilus and the CA3 area (Cabezas et al., 2012). Finally, BrdU staining experiments suggest EGFP+ cells in G42 mice are 15–28 days post-mitotic GCs, a time window shifted by about 10 days as compared to POMC-EGFP+ expression (Overstreet-Wadiche et al., 2006). We noticed a somewhat delayed EGFP expression in adult animals as compared to juveniles, which may represent a slight delay in the maturation process of GCs in adult hippocampus, although less pronounced than in POMC-EGFP mice (Overstreet-Wadiche et al., 2006; Zhao et al., 2006). This delay was also detectable in NeuN expression, which was more frequent in EGFP+ GCs in juveniles than adults. Overall, however, no significant distinction between EGFP+ GCs in juvenile and adult mice was highlighted in unsupervised cluster analysis including all molecular and physiological parameters. We conclude that GAD67-driven EGFP expression in G42 mice defines a rather specific, intermediate stage of GC differentiation throughout ontogenesis.

### Molecular and functional specificity of GAD67-EGFP expressing GCs

Single cell RT-PCR revealed a few remarkable differences between GAD67-EGFP expressing neurons and mature GCs, some of which may have a significant physiological impact. Regarding GABA synthesis, both GAD67 and GAD65 mRNAs were detected in EGFP+ GCs and, to some extent, in EGFP− cells as well. Yet, lack of GAD67 immunoreactivity in mossy terminals from mature GCs (Cabezas et al., 2012) suggests gene expression may not be sufficient to be detected at the protein level. Is GABA synthesized in EGFP+ GCs synaptically released? VIAAT expression in MFTs has been a rather controversial issue (Lamas et al., 2001; Sperk et al., 2003; Zander et al., 2010). Although we detected VIAAT mRNA at higher frequency in EGFP+ than adult EGFP− GCs, we were not able to detect VIAAT protein in EGFP+ MFTs by immunohistochemistry (Cabezas, Irinopoulou and Poncer, unpublished observations). Thus, whether GABA released from EGFP+ GCs during repetitive firing (Cabezas et al., 2012) involves vesicular exocytosis or, for instance, the reverse action of the GAT-1 transporter (Wu et al., 2007) remains to be explored. Interestingly, we detected mRNA for the vesicular glutamate transporter Vglut2 more frequently in EGFP+ than in EGFP− cells. In contrast, Vglut1 mRNA occurrence was similar in mature vs. EGFP+ GCS. Although Vglut1 is the predominant vesicular glutamate transporter at terminals from mature hippocampal principal neurons, transient Vglut2 expression during postnatal development has been observed in several brain regions (Boulland et al., 2004; Nakamura et al., 2005). Vglut2 may localize to a specific subset of presynaptic vesicles (Fremeau et al., 2004) and is usually associated with higher release probability synapses (Fremeau et al., 2004; Weston et al., 2011; He et al., 2012). In addition, Vglut2 was recently found to influence dendritic arbor maturation in hippocampal principal neurons (He et al., 2012). Thus, transient expression of Vglut2 in immature GCs may impact both their output dynamics and morphological maturation.

Tonic GABA currents have been suggested to play an important role in the migration, morphological maturation and synaptic integration of newborn GCs (Ge et al., 2006, 2007; Duveau et al., 2011). In mature GCs, these currents are carried predominantly by α4β2δ receptors (Glykys et al., 2008; Herd et al., 2008). Thus, genetic ablation of δ subunits results in a drastic reduction in tonic inhibition in these cells (Glykys et al., 2008). However suppression of α 4 but not δ subunit expression results in altered dendritic maturation of newborn GCs (Duveau et al., 2011), suggesting δ subunit may not play a major role in tonic currents in these cells. Our data from scRT-PCR support this hypothesis and show that δ subunit mRNA is considerably less abundant in immature than in mature GCs. Accordingly, gaboxadol, at a concentration specific of δ-containing GABAA receptors, potentiated tonic currents in mature, EGFP− but not in immature, EGFP+ GCs. Thus, we suggest contribution of δ subunit to GABAA receptors may be minimal in immature GCs. The subunit composition of extrasynaptic GABAA receptors in these cells remains unknown. Based on scRT-PCR data however, we suggest they may comprise α 5/γ 2 or α 4/γ 2 assemblies since mRNAs for all three subunits are frequently detected in immature GCs and are known to mediate tonic currents in other cell types.

GABA may activate GABAA receptors located not only on the somato-dendritic membrane but also along MFs (Kullmann et al., 2005). Activation of presynaptic GABAA receptors has been shown to modulate mossy fiber excitability (Ruiz et al., 2003) and influence the kinetics of APs and action potential-dependent Ca^2+^ transients in MFTs (Ruiz et al., 2010). These receptors likely comprise the δ subunit since they are sensitive to the endogenous neurosteroid tetrahydrodeoxycorticosterone (THDOC) as well as GBX. Our results predict the effects of GABA acting on MFs may be reduced in immature GCs as compared to mature GCs, or may be mediated by other receptor assemblies. Interestingly, we observed that GABA released from individual GAD67+ GCs was able to modulate mossy fiber excitability (Cabezas et al., 2012). This effect however persisted in the presence of bicuculline, was abolished by a GABAB receptor antagonist and was not observed in mature, GAD67− GCs. Thus, we suggest that distinct auto-receptors may be expressed along MFs from immature vs. mature GCs and trigger distinct signals that modulate mossy fiber excitability, perhaps in response to GABA released from different sources. The functional significance of GABAA vs. GABAB autoreceptor activation at mature *vs.* immature MFs remains to be fully characterized.

### Functional relevance of GAD67 expression in differentiating GCs

The GABAergic phenotype of dentate gyrus GCs remains a controversial issue (Sloviter et al., 1996; Walker et al., 2001; Gutierrez et al., 2003; Mori et al., 2004; Gutierrez, 2005; Safiulina et al., 2006; Uchigashima et al., 2007; Cabezas et al., 2012). GAD67 expression has been detected in GCs from young (<3 weeks postnatal) animals and under pathological conditions—such as epilepsy—in adults (Schwarzer and Sperk, 1995; Sloviter et al., 1996; Gutierrez et al., 2003). These observations appear consistent with GAD67 expression being restricted to immature GCs since (1) most dentate gyrus GCs are not fully mature at birth and undergo postnatal differentiation (Altman and Bayer, 1990), (2) the number of newborn neurons decreases with age (Kempermann et al., 2004) and (3) epilepsy is known to stimulate adult neurogenesis in the dentate gyrus (Jessberger et al., 2007; Parent, 2007). Thus, our results suggest that GAD67 expression in dentate gyrus GCs likely reflects the differentiation stage of individual cells rather than an age-specific or pathology-induced recapitulation of an immature phenotype (Gutierrez, 2005).

We have previously shown that GAD67 expression does not endow GCs with a full GABAergic phenotype (Cabezas et al., 2012). Although these cells may be able to release GABA that activates presynaptic receptors, no monosynaptic IPSCs could be detected in any of the post-synaptic targets tested. The timing of GAD expression in dentate gyrus GCs, however, coincides with differentiation stage 5, as proposed by Kempermann and collaborators (Kempermann et al., 2004), in which newborn GCs express immature markers such as DCx and calretinin. This stage represents a critical period for selection, survival and integration of newborn GCs in the hippocampal network (Dayer et al., 2003). Thus, we propose that GAD67 expression might be required for maturation of newborn GCs. Further work is needed to fully elucidate the functional relevance of this phenotype.

## Author contributions

Carolina Cabezas and Jean Christophe Poncer designed research; Carolina Cabezas performed all experiments; Carolina Cabezas, Theano Irinopoulou, Bruno Cauli, and Jean Christophe Poncer analyzed the data; Carolina Cabezas and Jean Christophe Poncer wrote the manuscript.

### Conflict of interest statement

The authors declare that the research was conducted in the absence of any commercial or financial relationships that could be construed as a potential conflict of interest.

## Appendix

**Table A1:** **PCR primers**.

**Targets**	**First PCR primers**	**Size (pb)**	**Second PCR primers**	**Size (pb)**
Prox1	Sense, 1590: ACACCATCTGAGCCACCACC	455	Sense, 1628: CACCCAGCACCGCAGAAG	167
NM_008937	Antisense, 2021: GGAATCTCTCTGGAACCTCAAAGT		Antisense,1773: GGGGTAGCGGGTGTAAAAGAAC	
GABA-a1	Sense, 1011: CAAGAGAGGGTATGCGTGGG	464	Sense, 1029: GGATGGCAAAAGCGTGGTTC	198
NM_010250	Antisense, 1273: CCAAATAGCAGCGGAAAGGC		Antisense,1205: TTGGGTTCTGGTGGTTTTGTCT	
GABA-a4	Sense, 1012: GCTGCTGTCAACTATTTCACCAA	366	Sense, 1064: TATCAAAGCCTCCCCCAGAAGTT	179
NM_010251	Antisense, 1360: AGGAGAGGGGGATGCTGC		Antisense,1221: GCTGGTCTTGCTGGAGTGATTT	
GABA-a5	Sense, 40: ACCCTCCTTGTCTTCTGTATTTCC	326	Sense, 155: TCTTGGACGGACTCTTGGATGG	175
NM_176942	Antisense, 346: TTGTTGAGAGGGAGGCGTTG		Antisense, 310: CGCAGCCTTTCATCTTTCCA	
GABA-g2	Sense, 902: GAAAATCTCTGCCCAAGGTCTC	394	Sense, 967: TTTGTGTTTTCTGCTTTGGTGG	264
NM_177408	Antisense, 1276: CAAAAGGCGGTAGGGAAGAA		Antisense, 1209: TCGGCAATCTTCAAAACAGCAG	
GABA-d	Sense, 954: CGTCTTTGTGTTTGCTGCCCT	252	Sense, 1011: CAGGAAGAAACGGAAAGCCA	127
NM_008072	Antisense, 1186: CCCTCCTTCTTTGCCTCCAC		Antisense, 1118: GACGACGGGAGATAGCCAACT	
GAD67	Sense, 529: TACGGGGTTCGCACAGGTC	598	Sense, 801: CCCAGAAGTGAAGACAAAAGGC	255
NM_008077	Antisense, 1109: CCCAGGCAGCATCCACAT		Antisense, 1034: AATGCTCCGTAAACAGTCGTGC	
GAD65	Sense, 99: CCAAAAGTTCACGGGCGG	375	Sense, 219: CACCTGCGACCAAAAACCCT	248
NM_008078	Antisense, 454: TCCTCCAGATTTTGCGGTTG		Antisense, 447: GATTTTGCGGTTGGTCTGCC	
VIAAT	Sense, 180: GGACATCCTGAAATCGGAAGG	322	Sense, 310: GCTGGTGGGGAGTTCGGG	118
NM_009508	Antisense, 501: GATGAGGATCTTGCCGGTGTA		Antisense, 408: GGAGGATGGCGTAGGGTAGG	
VgluT1	Sense, –113: GGCTCCTTTTTCTGGGGCTAC	259	Sense, –54: ATTCGCAGCCAACAGGGTCT	153
NM_182993	Antisense, 126: CCAGCCGACTCCGTTCTAAG		Antisense, 79: TGGCAAGCAGGGTATGTGAC	
VgluT2	Sense, 1: ATGGAGTCGGTAAAACAAAGGATT	346	Sense, 32: CGGGGAAAGAGGGGATAAAG	286
NM_080853	Antisense, 323: TGGCTTTCTCCTTGATAACTTTGC		Antisense, 296: CGGTGGATAGTGCTGTTGTTGA	
GAT-1	Sense, 1197: TACCGTGGAGGGCTTCATCA	340	Sense, 1249: CGCAATCGCCGTGAACTCTT	262
NM_178703	Antisense, 1519: AAACACGCCCGCCACAAT		Antisense, 1491: TGAAAAAGGACCAGCACAGC	
Dcx	Sense, 346: ATGGATGAACTGGAAGAAGGGG	469	Sense, 416: CCAAGAATGTCAACCCCAACTG	347
NM_001110222	Antisense, 793: CTGCATTCATTCTCATCCAAGG		Antisense, 741: AGGACCACAAGCAATGAACACA	
Tubb3	Sense, 54: CAAGTTCTGGGAGGTCATCAGC	319	Sense, 90: AGACCCCAGCGGCAACTATG	168
NM_023279	Antisense, 351: ACACTCTTTCCGCACGACATCT		Antisense, 237: CTGAATAGGTGTCCAAAGGCG	
CR	Sense, 83: TTGATGCTGACGGAAATGGGTA	265	Sense, 141: GCTGGAGAAGGCAAGGAAGG	151
NM_007586	Antisense, 327: CAAGCCTCCATAAACTCAGCG		Antisense, 271: ATTCTCTTCGGTCGGCAGGAT	
CB	Sense, 139: CGAAAGAAGGCTGGATTGGAG	426	Sense, 194: ATGGACAGAGAGATGATGGAAAAA	295
NM_009788	Antisense, 544: CCCACACATTTTGATTCCCTG		Antisense, 467: TCCAGCTTTCCGTCATTATTTG	
GFP	Sense, 185: TGACCACCCTGACCTACGGC	370	Sense, 301: TTCAAGGACGACGGCAACTAC	186
U55762	Antisense, 537: TGCTGGTAGTGGTCGGCG		Antisense, 466: GATGCCGTTCTTCTGCTTGTC	
ZnT3	Sense, 32: AGACCACCCGCCTAGTGAGT	464	Sense, 240: CGCTGTGTGCTTCATCTTCAT	178
NM_011773	Antisense, 495: GAAGGCCAGGTACAGGAGGAT		Antisense, 400: GTGCCAGCCAAAGGTCAT	
CLC2	Sense, 2034: GACATCTATCCGCTTCCAGGTG	484	Sense, 2063: AGGACTCGGGCTTCTCTGGA	405
NM_009900	Antisense, 2496: AATCCCAATGAGTCTGCCAATG		Antisense, 2446: GCAATGAGAAGATGGTGTGGGT	
NR2A	Sense, 813: GTTTCCATCGGGTCTCATTTCA	420	Sense, 862: CTGGAGGCAAGAGTGAGAGACG	275
NM_008170	Antisense, 1211: AAGGTGACAATGCTGAGGTGGT		Antisense, 1117: TCCCACTTGCCCACCTTTTC	
NR2B	Sense, 802: ACGGTGCCTTCAGAGTTCCC	428	Sense, 892: GCCATCATCACCACTGCTGC	239
NM_008171	Antisense, 1208: GGTAACGATGCTCAGATGGTCA		Antisense, 1110: CCACCCTCTCCCACTTCCTC	
GluR2	Sense, 37: CCTGTTTTATGGGGACTGATTTT	341	Sense, 87: AGGGGGGCTATTTCCAAGGG	191
NM_001083806	Antisense, 358: ATGACAAATGGATGCGTGCC		Antisense, 256: CGTAAAACCCAAAAATCGCATA	
GluR3	Sense, 365: GCTTTCCCACTGATGCCGAT	277	Sense, 415: TAAAGGGTGCCATTCTGAGTCT	212
NM_016886	Antisense, 618: TCAATCAAGTATCGTTTTTCCTGC		Antisense, 605: TTTTCCTGCCTTCTGTCCATTT	
KA2	Sense, 742: GACTTTCCCATCCTGCATCTG	488	Sense, 873: CTGTGAAGCCAGCACCTATCC	206
NM_008168	Antisense,1229: TGTGACAGGTTGATGTCCAGG		Antisense, 1078: CGGTCAGCCCGTCATACTCTA	
GluR6	Sense, 54: AGTCCTGCTCTGCTTGTTGTGG	347	Sense, 202: AGGACTCTGCTACCCAATACCACG	131
NM_001111268	Antisense, 379: AGCGGGTCTGTATGTGAGGAAC		Antisense, 313: TGTGAAGGACCGAAGATGGC	
mGluR2	Sense, 1142: TGGTCAATGCCGTGTATGCC	357	Sense, 1187: ACCGTGCCCTCTGTCCCA	196
XM_909627	Antisense, 1481: AGCGAGAGGCAGGGAGGG		Antisense, 1363: GCCCGCAGATAGGTGAAGAT	
mGlurR3	Sense, 1089: CCAGAACAAGAGAAACCACAGACA	313	Sense, 1142: GCAGCAACTATGAACAAGAATCCA	211
NM_181850	Antisense, 1380: GTTGTATCTTCCCATCCCGTCT		Antisense, 1330: TCTGCTCCTTTATTTGGGTTGAA	
NPY	Sense, 16: CGAATGGGGCTGTGTGGA	294	Sense, 38: CCCTCGCTCTATCTCTGCTCGT	220
NM_023456	Antisense, 286: AAGTTTCATTTCCCATCACCACAT		Antisense, 236: GCGTTTTCTGTGCTTTCCTTCA	
Intron	Sense, 8: CTGTCCCCCTTACGAATCCC	240	Sense, 16: CTTACGAATCCCCCAGCCTT	182
X51468	Antisense, 228: CCAGCACCAGGGATAGAGCC		Antisense, 178: TTGAAAGCCAGGGAGGAACT	
TI-VAMP	Sense, 4: GCCATTCTTTTTGCTGTTGTTG	418	Sense, 153: GTTTCATTACATCTGCCAAGACAG	179
NM_011515	Antisense, 398: CCATTATTCCTTTCAGTTCATCCA		Antisense, 309: CCAAAACACTTGAAAACTCGCTA	
KCC2	Sense, 58: GGCAACCCCAAAGAGAGCAG	299	Sense, 84: CATCAACAGCACGGACACGG	181
NM_020333	Antisense, 337: CGCAGGAAGAGGATGACACC		Antisense, 241: TTTTCCACCCTCATTATTTTCTGC	
NKCC1	Sense, 744: TTTTGAGGATGGCTTTGCGA	290	Sense, 810: TGCCGAGAGTAAAGGAGTTGTAAA	152
NM_009194	Antisense, 1014: CTCCTCCTCTCACGAACCCA		Antisense, 938: CCATTGCTATTACGACGACAGAGA	

*Position 1, first base of the start codon*.

**Table A2:** **Compared molecular properties of GAD67/EGFP expressing and non-expressing GCs**.

	**Group 1 EGFP+**	**Group 1 EGFP−**	**Group 2**
DCx	68.7%	63.6% ns	27.3%^**^^,^ ^##^
CB	59.4%	78.8% ns	86.4%^*^, ns
Cr	65.6%	75.7% ns	22.7%^**^^,^ ^###^
Tub3b	84.4%	87.9% ns	90.9% ns
GAT-1	78.1%	75.7% ns	45.4%^*^^,^ ^#^
VIAAT	34.5%	39.4% ns	13.6%^*^, ^#^
GAD65	31.2%	15.1% ns	18.2% ns
GAD67	56.3%	42.4% ns	45.4% ns
Vglut1	59.4%	78.8% ns	68.2% ns
Vglut2	56.2%	48.5% ns	9.1%^***^^,^ ^###^
GABAα1	53.1%	78.8%^*^	86.4%^*^, ns
GABAα 4	62.5%	66.6% ns	72.7% ns
GABAα 5	62.5%	81.8% ns	54.5% ns, ^#^
GABAδ	31.2%	42.4% ns	63.6%^**^, ns
GABARG2	43.7%	51.5% ns	59.1% ns
GluA3	28.1%	45.4% ns	72.7%^**^^,^ ^#^
GluA2	87.5%	69.7% ns	81.8% ns
mGluA3	15.6%	33.3% ns	4.5% ns, ^#^
mGluA2	56.2%	69.7% ns	59.1% ns
GluA6	75%	66.7% ns	63.6% ns
KA2	65.6%	81.8% ns	77.3% ns
NR2A	68.7%	84.8% ns	81.8% ns
NR2B	93.7%	96.9% ns	77.3% ns
ZnT3	37.5%	30.3% ns	31.8% ns
NPY	12.5%	24.2% ns	18.2% ns
Ti-VAMP	34.3%	42.4% ns	18.2% ns
KCC2	31.2%	36.4% ns	22.7% ns
NKCC1	71.8%	81.8% ns	50% ns, ^#^
CLC2	43.7%	27.3% ns	27.3% ns

Occurrence probability of molecular markers included in the sc-RT-PCR *n* = 32, 33, and 22 for group 1 EGPF+, group 1 EGFP− and group 2 GCs. Significance

* p < 0.05,

** p < 0.01,

*** p < 0.001, for comparisons with group 1 EGFP+ GCs and

# p < 0.05,

## p < 0.01,

### *p < 0.001 for comparison with group 1 EGFP− cells*.
